# Parents’ perspectives on a smartwatch intervention for children with ADHD: Rapid deployment and feasibility evaluation of a pilot intervention to support distance learning during COVID-19

**DOI:** 10.1371/journal.pone.0258959

**Published:** 2021-10-27

**Authors:** Franceli L. Cibrian, Elissa Monteiro, Elizabeth Ankrah, Jesus A. Beltran, Arya Tavakoulnia, Sabrina E. B. Schuck, Gillian R. Hayes, Kimberley D. Lakes

**Affiliations:** 1 Fowler School of Engineering, Chapman University, Orange, CA, United States of America; 2 School of Education, University of California, Riverside, CA, United States America; 3 Department of Informatics, Donald Bren School of Information and Computer Sciences, University of California, Irvine, CA, United States of America; 4 The Children School, Irvine, CA, United States of America; 5 Department of Pediatrics, School of Medicine, University of California, Irvine, CA, United States of America; 6 Department of Psychiatry and Neuroscience, School of Medicine, University of California, Riverside, CA, United States America; IRCCS E. Medea, ITALY

## Abstract

Distance learning in response to the COVID-19 pandemic presented tremendous challenges for many families. Parents were expected to support children’s learning, often while also working from home. Students with Attention Deficit Hyperactivity Disorder (ADHD) are at particularly high risk for setbacks due to difficulties with organization and increased risk of not participating in scheduled online learning. This paper explores how smartwatch technology, including timing notifications, can support children with ADHD during distance learning due to COVID-19. We implemented a 6-week pilot study of a Digital Health Intervention (DHI) with ten families. The DHI included a smartwatch and a smartphone. Google calendars were synchronized across devices to guide children through daily schedules. After the sixth week, we conducted parent interviews to understand the use of smartwatches and the impact on children’s functioning, and we collected physiological data directly from the smartwatch. Our results demonstrated that children successfully adopted the use of the smartwatch, and parents believed the intervention was helpful, especially in supporting the development of organizational skills in their children. Overall, we illustrate how even simple DHIs, such as using smartwatches to promote daily organization and task completion, have the potential to support children and families, particularly during periods of distance learning. We include practical suggestions to help professionals teach children with ADHD to use smartwatches to improve organization and task completion, especially as it applies to supporting remote instruction.

## Introduction

Attention Deficit Hyperactivity Disorder (ADHD) is the most prevalent childhood psychiatric condition, affecting nearly 1 in 10 children in the United States, with a profound public health, personal, and family impact [1]. ADHD requires comprehensive treatment, including child interventions (e.g., behavioral treatment, medication), parent training, and educational planning. Because ADHD is chronic and lifelong, maintenance, which requires improved executive function skills (e.g., organizational strategies) and substantial self-regulation, is needed to support initial treatment gains [2].

In March 2020, in the United States, California’s governor issued a stay-at-home order in response to COVID-19 [3]. In response to this order, schools physically closed and transitioned rapidly to emergency distance learning [4], including the school in which we were conducting research. The COVID-19 pandemic necessitated extensive school closures and reliance on remote instruction for a significant portion of the year. These necessary protective measures came with serious ramifications for teaching and learning, especially for the delivery of special education services. Specifically, the COVID-19 crisis was predicted as likely to be "particularly challenging for adolescents and even more so for those with ADHD" due in part to challenges with organization and increased risk of not participating in scheduled distance learning [5].

Evidence suggests that distance learning during the COVID-19 pandemic will likely amplify existing educational inequities and widen the achievement gap for disadvantaged students who may not have access to high-speed internet or high-quality computers at home [6]. Among the long list of recommendations provided to combat the amplification of educational inequities during such crises are to: (a) engage students and staff in the process of planning adjustments to curriculum and assessment to better consider the diverse circumstances of students, (b) heighten monitoring of learner well-being, (c) be sensitive to family stressors, (d) identify children and youth who are at high risk and plan interventions, (e) engage and empower families, and (f) access community resources [7, 8]. Golberstein et al. [9] noted the potential for "technology-enabled interventions" to "fill a substantial gap if demonstrated to be effective" while acknowledging the lack of current research in this area.

In 2015, 73% of adolescents in the United States had access to a smartphone, numbers that are only expected to rise [10]. Additionally, the benefits of Digital Health Interventions (DHI) may be felt even more profoundly by those most under-served by the existing healthcare system; low-income, youth, and minority status are all indicators of a greater likelihood of use of mobile phones for Internet access than more traditional methods [11]. Notably, researchers who focus on teens and mental health note that, for the most part, access to these smartphone technologies is relatively positive, regardless of what some limited studies might argue [12].

Smartphones can run sophisticated applications to record and analyze data and deliver educational messages to patients. Even in a pre-smartphone environment, simple text-based reminders delivered by mobile phones improved attendance at outpatient appointments and adherence to intervention plans [13–17]. By coupling the now near-ubiquitous smartphones with off the shelf smartwatch technologies, we can offer a sophisticated yet highly available alternative to existing, failing approaches to long-term maintenance. Although still a relatively small market compared to mobile phone penetration, smartwatches are increasingly a part of everyday life, particularly amongst younger people. In 2018, Deloitte’s global mobile survey indicated that 23% of Americans own a fitness band, and 13% own a smartwatch [18]. Major insurance companies have begun to prescribe and pay for smartwatches for long-term health management [19].

In this research, we explored how smartwatch technology using timing notifications can support children with ADHD during periods of distance learning. Specifically, the results presented here demonstrate the potential for a method to support children with ADHD using a smartwatch DHI. We hypothesized that the use of reminders would reduce the burden on parents to remind their children to attend virtual classes and help children develop more independent organizational and self-regulation skills. This research is part of a larger long-term project focused on developing and evaluating such DHI. In this paper, we present the results of the first phase of this work, a rapid deployment study in response to the COVID-19 stay-at-home order in California aimed at understanding and describing the feasibility of using a smartwatch as a DHI for children with ADHD. This study allowed us to get early feedback from the families, explore preliminary outcomes and provide the foundations for a future intervention.

## Rationale for DHI to support children with ADHD

### ADHD and organizational skills

Children’s ability to learn in large part relies on their ability to focus their attention where necessary, flexibly shift attention to important pieces of information, and sort out irrelevant pieces of information [20]. These cognitive abilities are central to executive functioning. Executive functioning is involved in the regulation of goal-directed behavior and includes abilities such as attentional control, strategic planning, organization, and cognitive flexibility [21]. Deficits in executive functioning in individuals with ADHD have been well-documented and include occupational problems with organization, time management, and planning difficulties [22].

Poor organizational skills might manifest as messy work environments for children with ADHD in school, misplacing or forgetting necessary school supplies, and inability to track assignments and due dates [23, 24]. Time perception has also been identified as an executive function [25], and cognitive studies of ADHD have identified deficits in time perception in individuals with ADHD [26]. It is evident that children’s differences in time management and planning lead to procrastination and incomplete assignments [27]. These types of impairments increase in severity as students progress through school [28, 29].

ADHD has also been associated with differences in emotion regulation skills. These skills include challenges in self-regulating the physiological arousal caused by strong emotions, difficulties inhibiting inappropriate behavior in response to emotions, difficulty refocusing attention after experiencing strong emotions, and disorganization of coordinated behavior in response to emotional activation [30]. Research from a longitudinal study suggests that differences in emotion regulation skills contribute to social problems in adolescents [31]. Children with ADHD have been shown to express greater negative effects during concept formation tasks associated with learning, which resulted in decreased use of effective problem-solving strategies and increased use of negative self-evaluations and solution-irrelevant statements [32].

These challenges among individuals with ADHD often persist into adulthood [33] and are associated with both symptom severity and impairment [34]. Because ADHD is chronic and lifelong, maintenance, which requires improved executive function skills (e.g., organizational strategies) and substantial self-regulation, is needed to support initial treatment gains [2].

Therefore, it is imperative to implement early interventions designed for children to help them to improve their executive functioning and self-regulation, especially with regards to skills related to their ability to succeed in school. A snapshot of the evidence-based interventions used to support students with executive functioning in schools includes (a) organization training [35], (b) self-monitoring and self-management to increase on-task behavior and academic productivity [36], (c) social skills training [37], and a variety of behavioral management strategies, commonly token economy systems [38]. Components of these interventions make implementation particularly difficult in a distance learning environment. Further, a wide range of difficulties faced by educators, school psychologists, and other service providers situated in schools have decreased the likelihood that these interventions are being implemented with integrity. There is also the likely possibility that interventions are not being implemented at all, or at least to a lesser extent compared to schooling before the onset of the COVID-19 pandemic [39].

### Mobile technology supporting schoolwork of children with ADHD

Although computing solutions have been investigated to support children with ADHD at school or online [40], mobile technology has been less explored. Currently, there are several available mobile applications that, although not targeting children with ADHD, can support academic work for post-secondary ADHD students (e.g., Google Calendar, Everbrite) [41] or their caregivers [42]. However, the literature supporting the effectiveness of these mobile applications for students with ADHD is scant.

Particularly, mobile technology in school settings has been used mainly to monitor behaviors of individuals with ADHD by sending schedule prompts via mobile phone to adolescents with ADHD to self-reflect [43] or teachers to assess children with ADHD behaviors [44]. These examples show that monitoring and assessing behaviors using mobile technology can help children with ADHD reflect on their actions more frequently and thus support them in their schoolwork.

Overall, research has shown that the proper use of prompts using mobile technology can support children with ADHD at school without distracting them. However, more research is needed to provide evidence of the effectiveness of those applications when used by students with ADHD in a school setting or while learning online at home.

### Mobile and wearables supporting ADHD organizational skills

Mobile and wearable technologies have been used to support children with ADHD to become more organized in family and home settings [45–48]. For example, MOBERO [47, 48], a tool to support families living with at least one child diagnosed with ADHD during their morning and bedtime routines, was tested for one month with 13 families. This study indicated that such technology could improve parent frustration and conflict levels around morning and bedtime routines. These improvements were stable for one month after they had MOBERO [47].

More recent research has explored how wearables can be used to support behavioral therapies for children with ADHD. For example, Garcia et al. [49] designed WRISTWIT, a wearable device (similar to a watch) targeted for children with ADHD between 8–12 years old designed to improve children’s ability to work independently by supporting the sense of time and attention. This tool uses a set of light-emitting diodes (LED) that display the time while gathering body movements by an accelerometer. WRISTWIT was designed following a user-center design approach involving children with ADHD and caregivers. Initial pilot-testing with children showed that their activity level was reduced using the device, which is promising but still limited.

These studies demonstrate how mobile and wearable applications have the potential to support daily functioning and life skills in children with ADHD. In our work, we have examined how smartwatches can be used as tools to support self-regulation and organizational skills in children with ADHD [50]. We have described design guidelines to develop self-regulation applications by using a hybrid approach to promote self- and co-regulation using a smartwatch for children [50]. We highlighted the advantages of smartwatches, such as their automatic tracking of behaviors, ability to deliver timely prompts, and the potential to deliver intervention discreetly using a mainstream device to avoid stigma among children and adolescents with ADHD [50]. As a first step and given the urgent need for supporting organizational skills during at-home learning, we deployed the first version of CoolCraig, a DHI aimed to support children with ADHD.

## Methods

This study was approved by the Institutional Review Board of the University of California, Irvine. Parents provided written consent for children’s participation, and children provided assent for their participation. The DHI was conducted in the Spring of 2020 and involved a connected system using an iPhone 8 (for the parent) and an Apple Watch 5 (for the child).

### Participants

Participants were enrolled in the study from March to May 2020. Participants were asked to indicate interest in involvement with the study via email. We then replied with an overview of the study and sent a consent form via email. We also offered to review and answer any questions via virtual communication. Both the parent and child were required to sign the consent form and send it back to the team before beginning participation.

We recruited ten children aged 10 to 15 years. Participants attend a nonpublic school serving children with ADHD and related behavioral challenges. This school provides daily behavioral health services, including group intervention to promote self-regulation and social skills. All participants presented with symptomatology characteristics of a diagnosis of ADHD according to the Strengths and Weaknesses of ADHD symptoms and Normal-behaviors (SWAN; [56]) and The Behavior Assessment System for Children- Third Edition (BASC-3; [57]; Table 1).

**Table 1:** Behavioral and emotional functioning: SWAN and BASC-3 PRS scores.

Questionnaire	Sub scale	Mean (SD)	Range
^**a**^ **SWAN**	Attention	-1.40 (.80)	0.11 to -2.33
	Hyperactivity/Impulsivity	-0.69 (1.20)	0.89 to -2.22
	Oppositional Defiant Behaviors	1.67 (1.41)	1.00 to -3.33
^**b**^ **BASC-3**	Externalizing	63.75 (14.06)	50 to 94
	Hyperactivity	59 (23.20)	11 to 83
	Aggression	54.13 (25.63)	7 to 97
	Conduct Problems	55.25 (24.35)	8 to 93
	Internalizing	59.25 (10.28)	48 to 79
	Anxiety	57 (12.14)	45 to 76
	Depression	63 (11.83)	50 to 81
	Somatization	53.75 (12.93)	44 to 79
	Behavioral Symptoms Index	68.13 (8.74)	54 to 82
	Atypicality	61.5 (9.23)	45 to 70
	Withdrawal	67.5 (13.56)	50 to 89
	Attentional Problems	68.5 (7.69)	55 to 78
	Anger Control	64.5 (11.33)	53 to 87
	Emotional Self-Control	60.88 (8.97)	48 to 76
	Executive Functioning	67.88 (8.56)	53 to 75

Note.

^a^ Strengths and Weaknesses of ADHD Symptoms and Normal-behavior (SWAN). On the SWAN, a negative score is indicated as a below average score (-3 being the lowest possible score/scale, 0 representing “average”, and +3 representing far above average). The three subscales of the SWAN (ATN = Attention, H/I = Hyperactivity/Impulsivity, and ODD = Oppositional Defiant Disorder) are calculated by averaging the answers (far below average = -3 to far above average = 3) associated with each subscale.

^b^ BASC-3 = Behavior Assessment System for Children, Third Edition; PRS = parent rating scale. The BASC-3 PRS scores displayed in this table are T-scores. On the BASC-3, a T score between 41 and 59 falls in the average range of development; scores between 60–69 are considered "at-risk," and scores above 70 are in the "clinically significant" range.

Enrolled participants completed the SWAN and the BASC assessment measures to screen for symptoms of ADHD and other mental health difficulties. ADHD symptoms include elevated attentional difficulties, elevated externalizing behavior problems, and elevated executive function difficulties, which are all observed in the present sample. The elevated mean scores in attention, hyperactivity and externalizing behaviors are representative of a sample of children with ADHD.

The parent-reported child race/ethnicity was Caucasian (80%), Pacific Islander (10%), Hispanic/Latino (10%). The sex ratio for ADHD ranges from 2:1 to 10:1 [51–53], and although recruitment was open to both males and females, the majority of the school’s population at the time of the study was male (>90%). Only parents of male children responded to express interest in enrollment. Therefore, all participants were male.

### Materials

Each participant received a smartwatch (Apple Watch Series 5) paired with a smartphone (iPhone 8). In southern California where this study was conducted, more than 66% of the population with a mobile device use an iOS product (https://deviceatlas.com/blog/mobile-os-popularity-by-us-state) allowing for more rapid adoption and a lower learning curve. Additionally, the Apple Watch provided the most accurate measure of heart rate [54, 55], prioritized the privacy of users, and is lightweight.

Each phone and smartwatch was associated with an email address and username that did not include any identifying information from the children. Prior to configuring the devices, we asked each parent to provide the height, weight, age, and wrist preference for each child to configure the smartwatches. We installed standard applications for intervention and data collection purposes on the devices (e.g., Weather, Heart rate, Calculator, Breathing, Activity). Participants were not allowed to install additional applications during the study.

To support home behavior intervention, distance learning, and daily transitions, we used Google Calendar to create a customized daily schedule for each child. The parents’ phone was synchronized with Google Calendar. Notifications were delivered 5 minutes before each event or class through both the smartwatch and the phone. In addition to gathering data on behavior, mood, physical activity, and sleep through the smartwatch, we developed and installed an application for the phone and an application for the smartwatch to collect data using the Healthkit framework (https://developer.apple.com/documentation/healthkit#overview).

### Procedure

Devices were delivered to participants’ homes using COVID-19 approved research protocols to ensure safe handling and appropriate physical distancing. Participants used the smartwatch daily for 6 weeks at the end of the 2019–2020 school year. One researcher acted as a "broker" between the research team and the parents to enhance the privacy of participants and COVID-19 protections.

After the delivery, a detailed manual with suggestions and instructions for use was sent to each family via email. Parents could ask questions and provide feedback via individual video conference meetings (using the Zoom platform) every two weeks. During these video conference sessions, the research team also briefly interviewed the children individually.

Participants were instructed to wear the smartwatch as much as possible, including weekends and at night. However, the research protocol allowed for flexibility in determining which times were best to wear the smartwatch and planning a strategy for daily charging. Parents chose to either use the paired phones themselves or provide them to their child; the smartwatch was completely capable of working as a stand-alone device.

After six weeks, once summer break began for the children, we scheduled individual video conference meetings with each parent to conduct final semi-structured interviews, and the "broker" picked up the smartphone and the smartwatch from the families.

### Data collection

Parents completed SWAN [56] and BASC-3 [57], prior to the delivery of the devices. The SWAN rating scale is a well-validated parent-report scale used to assess ADHD symptoms. The SWAN was constructed with the intent of capturing variability at the adaptive ends of attention and behavioral regulation as well as at the maladaptive levels of these dimensions. For example, the SWAN asks how well the child "listens when spoken to" (attention) or "awaits turn" (behavioral regulation). The SWAN includes subscales measuring attentional, hyperactive/impulsive, and oppositional defiant symptoms. All items were scored on a 7- point scale reflecting the degree of discrepancy from average behavior: -3 = far below average, -2 = below average, -1 = somewhat below average, 0 = average, 1 = somewhat above average, 2 = above average, and 3 = far above average. Previous work has demonstrated the validity, reliability, and utility of scores derived from the SWAN rating scale to measure symptoms of ADHD [58–62].

The BASC-3 is a standardized assessment designed to be used in school and clinical settings for assessing positive (adaptive) as well as negative (clinical dimensions) behaviors related to behavioral and emotional problems experienced by children and adolescents. The BASC-3 Parent Report Scale (PRS) provides the parent’s ratings of their children. The BASC-3 PRS has four main composite scales: Adaptive Skills, Behavioral Symptoms Index, Externalizing Problems and Internalizing Problems. In this paper, we report the composite and subscale scores that make up the Behavioral Symptoms Index (BSI), Externalizing Problems (EXT), and Internalizing Problems (INT). We also report Anger, Emotional Control, and Executive Function scores due to their relevance to the behavioral targets for our intervention. Previous work demonstrates the psychometric strengths of BASC-3 scores, including high internal consistency as well as high inter-rater and test-retest reliability [63].

To better understand the parents’ perspective, receive early feedback from them, and explore preliminary outcomes of the DHI, after six weeks, we conducted semi-structured interviews with 7 of the 10 parents. These interviews provided qualitative data on the adjustment to the DHI for both the children and the family. Three parents chose not to participate in the interviews at the time as they described feeling overwhelmed by the circumstances of the pandemic. Interviews addressed topics around smartwatch wearing behaviors, novelty effects, concerns, advantages and disadvantages of using the smartwatch, and potential impact on organizational skills and self-regulation (see S1 File).

During the six weeks, we collected a sample of smartwatch data with the developed app. We collected samples of steps (n = 72,200), activity energy (n = 116,400), standing time (n = 2,700), heartrate (n = 32,900) and sleep (n = 435). Collection of data samples followed Healthkit framework standards, and data were stored in a deidentified and encrypted database.

### Data analysis

The research team used a mixed-method approach to data analysis [64]. We combined qualitative analysis of the interview data with quantitative analysis to examine the log stored from the smartwatch data.

All tele-conference interviews were conducted via the Zoom platform and audio recorded only. The interviewing researcher documented administration notes during and after the interviews. Six of the seven interviews were also transcribed verbatim (one interview had low recording quality and could not be reliably transcribed).

The first two authors coded the transcripts using an inductive approach based on a variation of thematic analysis [65], the most common data analysis method used in qualitative work [66, 67]. During the first stage of analysis, each researcher read the transcripts independently and identified repeating ideas or patterns of ideas as themes. Collaboratively, the investigators labeled themes and constructed initial codes. During the second stage, the research team conducted focused coding, whereby the major and minor themes and their properties were described, categorized, and organized into a codebook. Each coder then independently coded participants’ comments utilizing the codebook, which was revised as coding progressed. Regular meetings were held to discuss the codes assigned to comments for each transcript and to arrive at a consensus. This collaborative, reflective process allowed for the expression and discussion of multiple perspectives and prevented the personal or disciplinary biases of a single researcher from excessively influencing the findings. Coders corroborated 3 major themes and 15 minor themes from the interviews. Coders chose 2 out of 7 total interviews to corroborate coding schemes (i.e., test reliability) and were in 97% agreement across codes.

To estimate how many days children wore the smartwatch, we computed the number of days we collected any kind of data from each participant. (i.e., steeps, heart rate, activity energy, and stand minutes). Similarly, to estimate the amount of time the children wore the smartwatch each day, the sum of time between the samples obtained was calculated. According to the HealthKit framework, the smartwatch only records these data when the users wear the smartwatch. We used the NapBot—Auto Sleep Tracker application (https://apps.apple.com/us/app/napbot-auto-sleep-tracker/id1476436116) to collect how many days children wore the smartwatch at night. Finally, we recorded total steps children accomplished per day using the Health app from the iPhone. All of these data follow the sample windows from our app, which may be different from the raw data obtained directly from the Health application of the iPhone. For each set of data, we computed the descriptive statistics.

To represent our results, in the following subsections, we present the key finding under each main theme using verbatim quotes from participants to illustrate those findings [68] and quantitative data captured from the smartwatch.

## Results

Major and minor themes identified in the analysis are shown in Table 2.

**Table 2:** Major and minor themes.

Major theme	Minor theme	Description
Smartwatch Adoption	Wearing Behaviors	How often the child wore the smartwatch and any patterns to that wearing (e.g., daytime only versus nighttime). In particular, whether the child understood the smartwatch as a tool for school only.
	Familiarity	Whether and how child and family had used smartwatches previously, and the presence of any indication of “novelty effects” of the technology.
	User and Sensory experience	Whether the child experienced sensitivity issues. Reports related to the user experience about the smartwatch functionality.
Parent Perceptions	Initial expectations	Parents’ expectations about utility of the smartwatch (e.g., reminders will be useful for scheduling).
	Concerns	Parents’ reported concerns about the smartwatch (e.g., child losing or breaking the smartwatch).
	Roles	Parents’ perceived roles in the study, included reminding child to wear the watch or actively charging and syncing the watch themselves.
	COVID-19’s impact	Parents’ reported feelings about COVID-19’s impact on the feasibility of smartwatch use.
Potential Impact on Child Behavior	Attention	Any indication that the smartwatch inappropriately distracted the child, or helped them to attend to something they were meant to attend to.
	Organizational Skills	Indications that the reminder system associated with the smartwatch impacted children’s organizational skills (e.g., class attendance).
	Self-Regulation	Indications that the smartwatch impacted the child’s ability to regulate their emotions.
	Physical Activity	Indications that the child used and/or understood the physical activity indicators on the smartwatch (e.g., number of steps taken, heart rate).

### Smartwatch adoption

#### Wearing behaviors

Only one parent reported that his child had worn the smartwatch every day, and all day:

*He wears it 24 hours a day*. *He never takes off*. *He loves it*. *Even in the pool*, *I was freaking out*. *I’m like*, *"Oh my gosh*, *it’s not waterproof*!*" He’s like*, *"No*, *no*, *it’s good*. *It’s good*. *I need to keep it on*.*"* Parent 006

Two of the parents reported that their children wore the smartwatch during the day but not during the night. Some of the children reported that sleeping with the smartwatch was uncomfortable. Two parents described their children wearing the smartwatch only during the morning to support distance learning.

*When he wore it*, *he typically [wore] it during the day*. *He didn’t wear it as much at night because for whatever reason*, *he said it bothered him to sleep with it*, *but he did wear it through the day*. Parent 001

Two parents were unsure about when their children wore the smartwatch describing their children as independent in their wearing behaviors.

**Fig 1:**
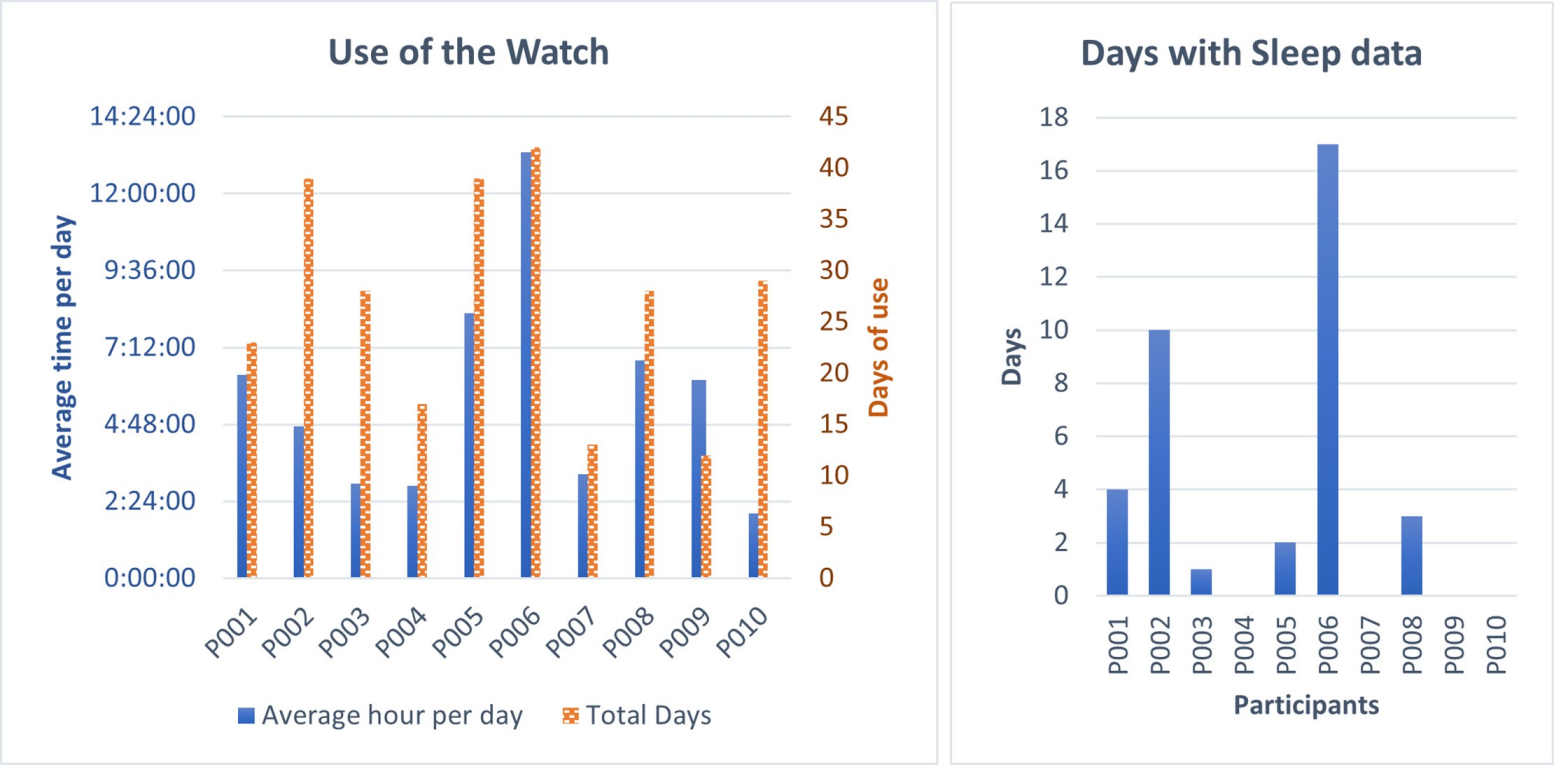
Use of smartwatch. A bar graph showing the estimation of the number of days (y-axis left), and average time (y-axis right) each child worn the smartwatch at any time of the day (left), and at night (right).

Our quantitative data confirm the parents’ observations; on average, children wore the smartwatch 64.3% of the days. One of them worn the smartwatch every day, and only three of them worn the watch less than 50% of the days (Fig 1-left). As parents also observed, we only recorded sleep data from 6 children, but for 8% of the days on average (Fig 1-right). We also estimated that children wore the smartwatch on average 5.66 hours per day (SD = 3 hrs.; Max time of use in one day = 21.66 hrs.; Min time of use in one day = 0.03 hrs.), two children used the smartwatch more than 8 hours per day on average, four of them between 4 and 6 hours, and the rest of them 3 or fewer hours (Fig 1-left).

Overall, children were willing and able to wear the smartwatch regularly during the day while they were participating in distance learning.

#### Familiarity with technology and novelty effect

All parents reported that their children were excited when they received the phone and smartwatch. Children did the unboxing, charged the device, and then spent time exploring the technology.

Although five children owned a phone, none of them had a smartwatch previously, and only one child used a regular watch. Only two of the participating families reported that one of the parents owned a smartwatch, and one of them reported that their son had asked for a smartwatch previously. Another family also reported that their child wanted a smartwatch even though no one in the family had one.

The child of one of the parents who already owned a smartwatch was thrilled to learn his was a newer version than his mother’s:

*He was very excited… We opened it right away*. *We tried to put it on*, *we turned it on*, *so it was all*, *‘Oh*, *look at this*. *Mine is better than yours*.*’ ‘I got the newer [watch]’* Parent 003.

However, all parents reported that after one week of wearing the smartwatch, the novelty effect passed, and children started to use it just as an assistant for their schedule or as a regular watch. Thus, the "cool" gadget was transformed into a "useful" tool:

*And then [after one week of wearing the smartwatch] just becomes a tool*. *No longer fun*, *it just becomes a tool*. *…* Parent 010

**Fig 2:**
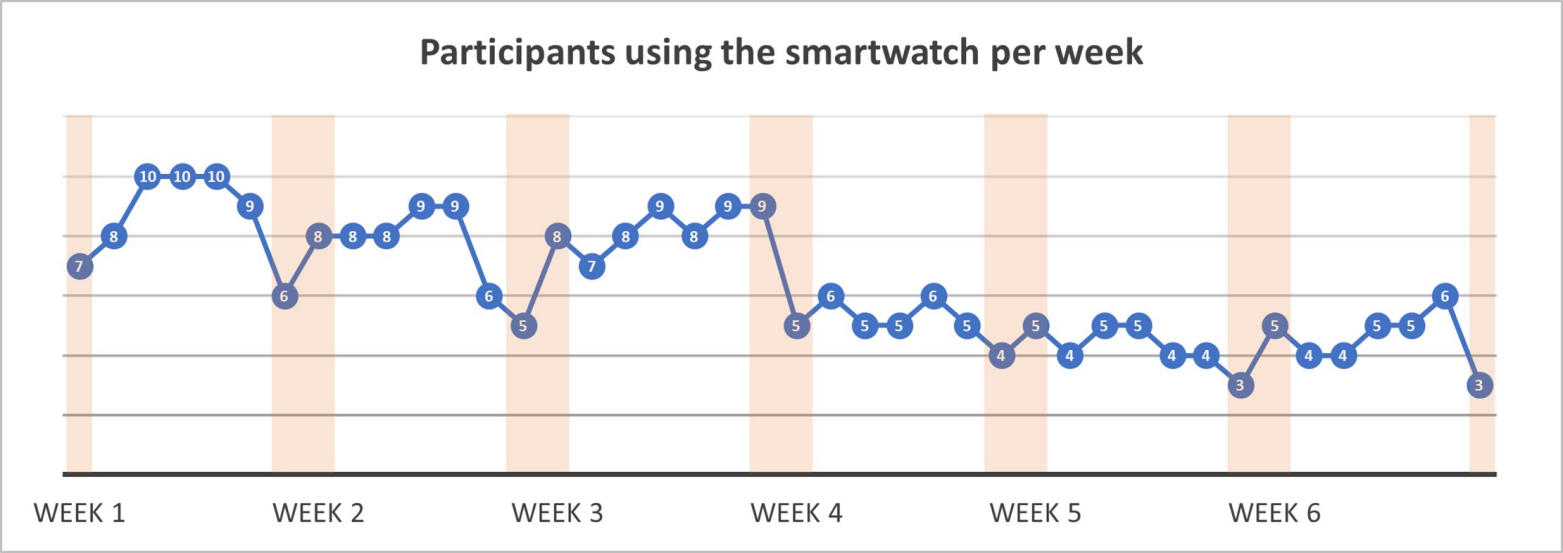
Participants wearing smartwatch per day. Number of participants who wore the watch each day. Orange bars represent weekends.

Moreover, our smartwatch logs show that most children wore the smartwatch during the first three weeks of the study (Average = 8.14). After that, average use decreased by half (Average = 4.66; Fig 2). Children wore the smartwatch during weekdays much more than during the weekends (Fig 2). This may indicate that they found the tool more useful during distance learning than during the weekends.

Given the particularities of conducting a study such as this during homeschooling in a pandemic, it is difficult to generalize from this result, but it is important to understand how long children might remain engaged with such tools. The novelty effect results are consistent with previously reported research [69]. Additionally, while many other studies show abandonment of wearable devices once the novelty effect wears off (e.g., [70]), the children continued to use the smartwatch as a tool but not as regularly as in the beginning.

#### Parent reports of children’s experience

Five of seven parents reported that their children enjoyed wearing the smartwatch, and most of them described their sons as "techy" children.

*I think he was excited about [the smartwatch]*, *because [he]’s a digital guy*. *He loves his iPads*, *Prime books*… *He’s technologically smart*. *And so*, *it’s just a part of that*. *So it’s just adding another feature to him and I think he was excited about that…* Parent 002

One parent said that his child was initially resistant to the smartwatch but became more willing:

*He was resistant to put it on*, *and then we put it on*. *He played with it for a little bit*, *and then he said he loved it…* Parent 004

One of the main advantages parents and children reported for the smartwatch was its portability and accessibility; for example, one parent noted:

*To be able to do this (look at his wrist)*, *instead of (trying to find the phone) … Just look at the watch and have things*, *instead of pulling out a phone*, *because he is very clumsy*. *And so*, *he tends to drop things*, *and break things*, *and it was nice to be able to just have that on his wrist…* Parent 006

Other parents noticed that their children preferred their phone due to sensitivity concerns and limited functionality. As previously reported, some children with ADHD have tactile sensitivities [71, 72], and may be reluctant to wear certain accessories. Encouraging their child to wear a smartwatch was a challenge for these two families:

*He’s not used to wearing things on his body*, *so the watch strapped to his wrist*, *I think is a different sensation to him*, *a different feel…* Parent 010

One child liked the smartwatch but not the sensation of wearing it, so he tried to wear the smartwatch loosely, which affected the availability of some functionality as well as our ability to collect physiological data:

*And he said*, *no*, *and in fact*, *he was keeping it really loose*, *for example*, *that’s why he would take it off and on…* Parent 003.

On the other hand, some parents also reported that the lack of ability to download and install additional apps that the children might have preferred was a factor that affected how they used the smartwatch.

*If he could have talked to his friends on it or something*, *I think he would have been a little bit more into it*, *but I think that is what sort of held it back…*. *And again*, *that’s where I think when I asked him*, *"Why aren’t you wearing it*?*" And he’s like*, *"Well*, *it doesn’t do anything for me*.*"* Parent 001

Overall, most children were excited to wear the smartwatch. Some wore it all day, and others used it as a "tool" to assist them with organization and task compliance during distance learning. Resistance to wearing the smartwatch consistently was primarily due either to tactile sensitivities or frustration in not being able to add features to the smartwatch.

### Perceptions of the impact on child behavior

#### Organization

During this six-week phase of the study, the smartwatch was programmed to synchronize with the child’s school schedule during distance learning. The smartwatch provided reminders 5 minutes before the children needed to attend class. When parents were asked whether the smartwatch seemed to affect their child’s organizational skills, four of them described already seeing an improvement:

*Especially more of when we were doing school at home*, *the distance learning*. *To have that on there*, *and to send him all that*, *Okay*, *you have math in 10 minutes*, *or you have a 5-minute break to do that*. *That was amazing*. Parent 006

*I think he was more aware of the schedule and kept him more accountable for doing it on his own*. *And it also showed me that he’s not only capable of doing it*, *following a schedule like that*, *but also he has a desire to*, *and that distraction and other things that prevented him from being on time to school was literally that just being distracted*. *So the watch allowed me to see that he actually wanted to be on time*. *He just needed a tool to help him get back*…Parent 004

Two of the parents had not yet noted noticeable improvements, and one parent thought her child may have stopped using the smartwatch because he found the notifications annoying.

*I think that that was one of the reasons why [child 001] stopped using it is he didn’t want to be buzzed at that time*, *but well*, *you got to get to class*. *So he felt that he could take it upon himself to get that done*. Parent 001

Prior studies have consistently indicated that children with ADHD thrive in structured environments [73–75]. These results indicate that the smartwatch can mimic some of the structure of those environments even in less structured contexts, like the home during distance learning.

#### Self-regulation

After interviewers provided a brief definition of self-regulation, they asked parents whether their children’s self-regulation skills were improved with regular use of the smartwatch and which features they perceived as having a positive effect on self-regulation. One parent found the breathing application and the emotional awareness and self-regulation application helpful:

*There was a notification that would come up every so often for my son that would say breathe*, *and he would breathe*…*He took it as a literal direction*. *And so he was like*, *"I guess*, *I’m not breathing*.*"*… *I think he was having a good time with that and liked it*. *And he also liked the little mood faces*…*So anyways*, *yeah*. *I think he engaged in that quite well*… Parent 004

Some parents described the smartwatch as having the potential to promote self-regulation but that this potential was not addressed by existing applications.

*… I think it can make them stop and do it*… *Where I can just go*, *I need to do this for myself*. *And they might not know that*. *He might not know that*. *So*, *if that can prompt him*, *or even send a signal to be like*, *"Hey*, *hold on*, *think about this*.*" Or maybe*, *stop*, *breathe*, *take a moment*, *think about whatever you’re going to do*, *or what you were doing*…Parent 006

*I think it kept him more conscientious of how he was reacting to*, *maybe*, *situations*. *I guess it just kind of reminded him of*, *"Oh*, *hey*, *I need to watch certain things and remain calmer*.*"*…. *I think it would help if they had other detection things*. *I think they have heart rate*, *but when he starts to escalate into something*, *he tends to get overheated*, *so it would be kind of neat if they had a temperature thing*…Parent 008

Few works have studied self-regulation strategies for people with ADHD specifically during distance learning, despite self-regulation being fundamental to developmental stages and trends towards increased online and home-schooling. Our results indicate parents recognize the potential for such an application, and some parents suggested the utility of the self-regulation application could be improved after further development of additional components to measure the physiological effects of emotion.

#### Attention

Given concerns that wearable devices could be a distraction, parents were asked explicitly about any attentional issues that could be attributed to wearing the smartwatch regularly. Four parents reported no distraction caused by the smartwatch; one reported she was not sure if the smartwatch distracted her child; and one parent said her child seemed distracted only at the beginning. Parents did, however, have to adapt existing rules to now include the smartwatch.

*When it was time to put it down*, *or charge it*, *or whatever*, *it was*. *Okay*. *And I would tell him*, *"We’re not doing phones*, *and now you say you’re not doing phones and watches at the dinner table*.*" And it was okay… It wasn’t too distracting*…Parent 006

Only one parent reported the smartwatch was distracting for her child due to the tactile sensitivity experienced by her child.

*… it wasn’t drastic*, *but I know that with him*, *he was definitely not used to wearing it*. *So I’m sure that it distracted him a little bit to have this new thing kind of strapped to his wrist*, *which he’s not used to*…Parent 010

One of the parents described the features of the DHI as contributing to the experience of it not being a distraction, specifically the lack of downloading additional applications:

*I think if he had the ability to download a bunch of stuff and play with it*, *it probably may have been worse*, *but I didn’t prevent him from doing that*. *He just didn’t*. *So*, *you know what I mean*? *… I think I agree with that from that standpoint*, *that was fine*. *But if a child is out in the real world*, *and they have a phone*, *and they have their watch*, *they’re probably going to download things*, *so that’s*, *in my mind*, *more kind of real life*… Parent 004

Our results indicate that concerns about DHI being distracting to children with ADHD may be overblown, but any new intervention should carefully consider what features and functionality to make available as well as the possibility of additional applications being added to the device, which may potential detract from the effectiveness of the DHI

#### Physical activity

Parents described substantial difficulties in promoting physical activity in the context of a state-wide stay-at-home order.

*There was no physical activity*, *especially the last*, *I don’t know*, *month or so of the shutdown*. *He just stepped down*. *The thing is*, *I think*, *you’re battling*, *the challenge of the kids being shut at home during this time*…Parent 001

Some parents described the smartwatch as positively impacting motivation around physical activity, with one parent noting that his son rode his bicycle more often and for longer distances in response to feedback from the smartwatch.

*Actually*, *he’s been riding his bike a lot*, *and he’s been timing it on the smartwatch*. *He rode 10 miles the other day*. *And I think that he enjoyed looking at it and saying*, *"Okay*, *I did this*. *I exercised for this long*.*" And then*, *the next day*, *he wanted to beat that ride*. *So*, *actually*, *I really liked that about it*. *Just physically being able to see it*, *instead of just*, *Oh*, *I rode my bike*. *He’s like*, *"I rode for 10 miles*!*"*…Parent 006

**Fig 3:**
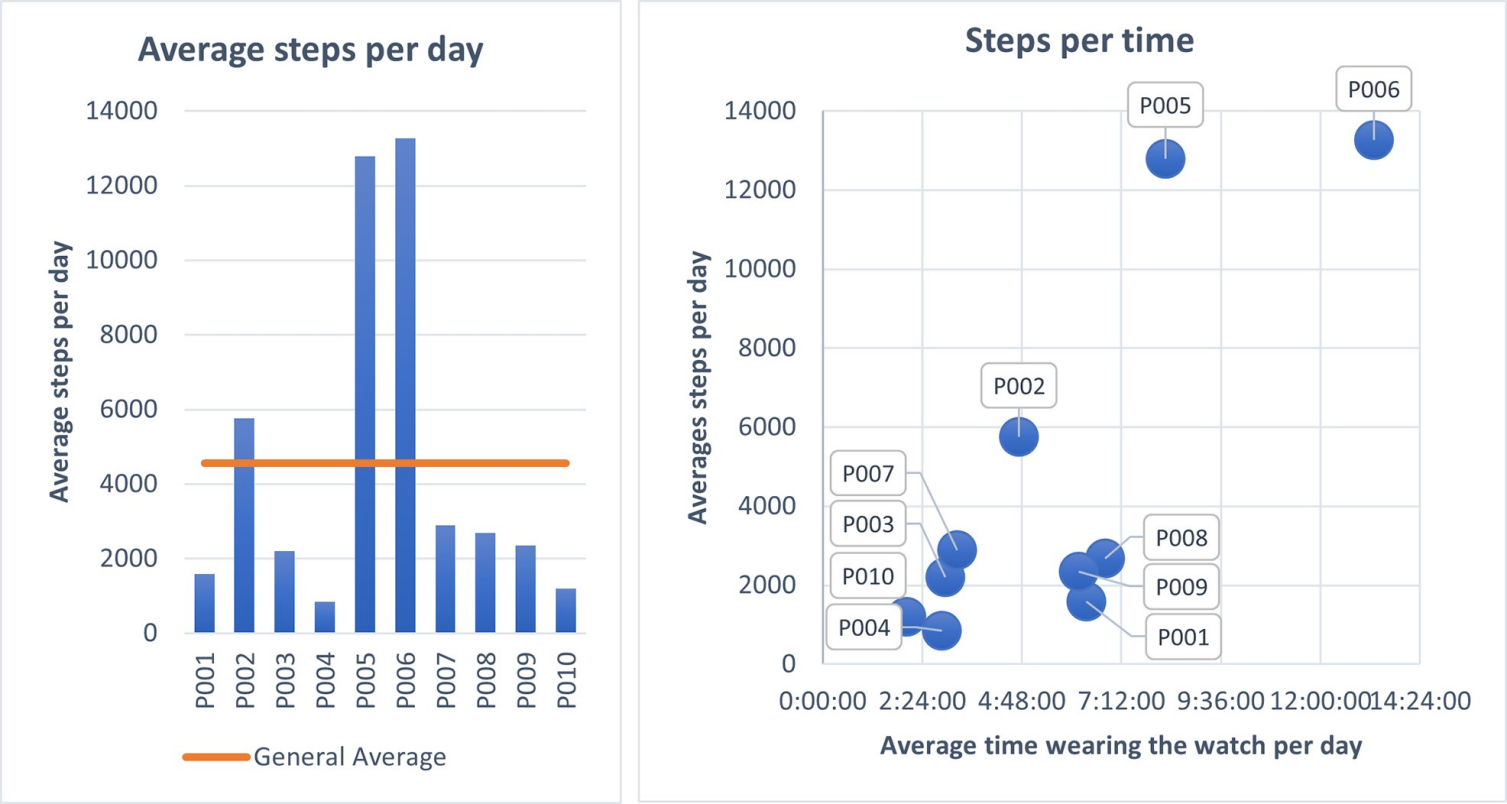
Steps. Average steps participants completed per day (left), and the comparison of steps per day and time each child wore the smartwatch (right).

Analyzing the steps children took each day according to the smartwatch data, we observed that only two children were meeting the widely recognized goal of 10,000 steps per day on average, (P006 and P005); one logged more than 5,000 steps per day on average (P002). The remained or children logged fewer than 3,000 steps per day on average (Fig 3-left).

Considering the average steps counts and the duration of time participants wore the smartwatch, our data revealed that steps and wear-time were correlated with those with the highest step counts also wearing the devices the most (P003, P004, P007, P010; Fig 3-right), and vice versa (P005, P006, Fig 3-right). Thus, it may be the case that the children not logging steps were getting sufficient physical activity, but that it was not recorded. However, one participant (P002; Fig 3-right) wore the device less than most of the other participants and still logged above 5,000 steps per day on average. Further research and analysis, perhaps coupled with self-report regarding daily bouts of physical activity or exercise, should be conducted to understand these behaviors better.

Some parents did not observe any changes in physical activity behaviors but did see more awareness in their children about how physical activity affects their physiological data:

*So during the COVID-19*, *in the month that we really stayed home*, *we were doing exercises in our garage through our gym*, *actually*. *We were posting 45 minutes exercises*, *like hard ones*. *And we would do it together every day*. *And so*, *I was like*, *"You can use it*. *And you can see your heart rate*, *how many calories*, *and you can also challenge yourself into it*.*"*…Parent 003

*He found it very interesting*… *we have a trampoline*, *so he would actually get on the trampoline with the watch because he wanted to see his heart rate*… Parent 010

Smartwatches are popular as fitness devices, and these results indicate that children with ADHD can use them in this context as well.

### Parent perceptions of a Digital Health Intervention (DHI)

#### Initial expectations and concerns

Parents expressed different interests in using technology to assist their children. Most of them expected that notification and reminders would be useful, particularly to support children during distance learning,

*My expectations were that it would help him like those reminders during the day*. . . .Parent 003

*… since we’re doing distance learning here at home*, *I thought it will be very helpful for him to remind him*, *"Hey*, *you have a class coming up*.*" Because I get exhausted*… *So I thought if the watch could help me keep him on task for his classes*, *oh my God*, *what a relief for me*… Parent 010

For most parents, their expectations were fulfilled. Two parents who expressed specific expectations, such as motivating their child to do more exercise or allowing them to monitor their children’s sleep patterns, found these expectations were not met in this phase of the study.

*He needs to start exercising more as COVID has kind of made him very sedentary*, *so I think I want to use it for tracking his steps and activity and maybe setting some little reminders*. *"Okay*, *you need to get up and do 20 jumping jacks*,*" or something like that…* Parent 008

*Well*, *I think the only thing is I really hoped that he wore it at night because I was actually kind of excited about being able to see his sleep pattern*…Parent 001

One of the parents said that she did not have any concerns at all,

*I didn’t have any concerns*. *I really didn’t*…Parent 001

Major concerns expressed by parents at the start of the study were that their children would break the smartwatch (n = 2), get distracted (n = 2), lose it (n = 1), forget to charge it (n = 1), take it away (n = 1), use it improperly (n = 1), or not be able to wear due to the tactile sensitivity (n = 1). With the exception of the concern related to tactile sensitivity, the remainder did not come to pass during the study period. Children did not break or scratch the watches. Most of them charged the watches daily and used them in the prescribed manner.

Our results show that parents were positively surprised by the ability of their children to be responsible for this electronic device. Although not all their expectations were accomplished in this phase, the majority of their concerns were satisfactorily overcome.

#### Parent roles in supporting the DHI

During the six weeks of use, six parents reported that they reminded their children to wear the smartwatch. Two of them ascribed this behavior to a need to remind or the child would not adhere to the intervention. One parent even went so far as to check whether reminding was necessary:

… *It wouldn’t have happened if I didn’t help him*. *And in fact*, *some days I didn’t help him purposely to see what he would do*. *And he remembered halfway through the school day*…Parent 004

The other four parents who reported that they needed to remind their children were less confident their children would not adhere but participated in reminding behaviors anyway. For example, one parent described checking her son’s behavior before prompting him:

*I tried to make sure he was wearing it*. *That for me was the big thing*, *is periodically going in and checking to see if he had it on*…Parent 001

One parent reported that she did not remind her son at all, as he was very independent, responsive, and engaged with the smartwatch.

Parents were accustomed to giving their children reminders and indicated that reminding them to use the smartwatch was not difficult or cumbersome. Most took it as part of their routine, and some of the parents even said they preferred reminding them to use the smartwatch as opposed to having to provide all the task reminders that the smartwatch now provided.

*… if it’s not the watch it’s me that has to remind him to get on*. *So it was actually a good tool*…Parent 004

*Well*, *I kind of figured that because of who he is at this point in my life*, *that’s kind of what I do every day with everything related to him*. *I’m always stepping in and having to give him that little push to do something*…Parent 010

While parents often reminded their children to wear the device daily, children quickly learned how to use and maintain the device adequately, and parents appreciated how the smartwatch would help with prompting throughout the day, reducing the burden on parents to provide all of the prompts necessary to help children participate in distance learning and complete other tasks.

## Discussion

### Use of DHI during distance learning in response to COVID-19

The COVID-19 pandemic necessitated extensive school closures and reliance on remote instruction for a significant portion of the academic year. These necessary protective measures came with serious ramifications for teaching and learning, especially for special education and behavioral health service delivery. Recognizing that the stay-at-home order presented new significant stressors for adolescents with ADHD and their families, we adapted our ongoing work to study how DHI might support families during distance learning.

In this timely and significant research, we explored how a smartwatch DHI could support children with ADHD and their families during distance learning. Overall, parents reported that the smartwatch intervention was acceptable to parents and children and was feasible to implement, even during pandemic-related stay-at-home orders. Parents reported that the smartwatch did not distract children from participation in their regular activities and that timing notifications were useful to promote organization and task completion. Parents described the smartwatch as having the potential to support children’s self-regulation and to promote physical activity by motivating the children through feedback on their activities.

### The potential for DHI to support parents and children

Parents are arguably some of the most adversely affected individuals when it comes to school closures and the employment of distance learning in response to COVID-19. School closures brought greater responsibility for parents, forcing them to consider their children’s needs in a novel and comprehensive way. Parents were abruptly tasked with considering their children’s needs for structure (e.g., regular wake-up and bedtimes), establishing an educational schedule, including physical activity and leisure time, helping their children to stay connected with others, and explaining the COVID-19 pandemic and helping their children to stay calm and reduce their anxiety [76]. Parents were likely concerned about their children falling behind in school, what sorts of questions to be asking school staff, and how to get children back into "school mode" [77]. As school began to reopen under different models (i.e. staggered start, hybrid models, temporary outdoors, reduced class size) parents again were forced to make difficult decisions with respect to their own and their children’s safety that will likely have social, behavioral, and academic repercussions [78, 79]. These demands shift continuously, requiring rapid responses from parents to adjust to the latest developments. In light of these challenges, our study focused on parents’ perspectives of the potential for DHI tools to support them and their children. We were interested in whether parents perceived the smartwatch and phone application to be useful during this especially stressful time, and as the results indicated, parents did perceive the tool as useful and provided feedback with implications for the future development of the DHI, which we are now developing.

### Lessons learned

Although this was a pilot study, the feedback provided by the parents and analysis of the outcomes provide valuable lessons about the feasibility of adopting wearables as DHI. Additionally, our experiences provide key insights into how to evaluate longer and large interventions in the future (for more details on study adaptation, financial considerations, and for data collection decisions made in response to the pandemic, please see [80]).

Our results indicate that children with ADHD can wear and charge a smartwatch regularly, and the proper delivery of prompts can support children’s organizational skills without necessarily being distracted. However, improvements on smartwatch bands and alternative ways of using the wearable should be explored to better support children with sensory differences. Although this pilot study was conducted during the stay-at-home order, some insights into the potential of learning and reflecting about the physical activity data available on the wearable should be further explored.

Overall, this pilot study showed that it is feasible to use wearables as a DHI and that such approaches have the potential to support families of children with ADHD. To provide an in-depth understanding and assess the efficacy of this type of intervention, we recommend conducting a Randomized Controlled Trial with a pre and post-intervention evaluations. During the intervention, wearable logs should be collected and labeled with events that allow the development of behavioral models. Regular meetings with the families will also allow researchers to collect feedback aimed to gather a better understanding of how DHI should be updated, improved, and developed in the future.

### Limitations and future directions

This was a necessarily limited pilot study, involving only ten families over six weeks in an unprecedented context. Thus, certain limitations, such as lack of formal diagnostic assessments at enrollment and the lack of a control group, were unavoidable; these limitations will be addressed in a future randomized trial. Future iterations of this study will involve the collection of data using standardized rating scales to yield quantitative scores, which allow an individuals’ score to be compared to a normative sample. These rating scales will be completed by parents at baseline and post-intervention so that the significance of changes in scores can be quantified. The standardized rating will be correlated with the smartwatch logs that may allow us to create better machine learning models to understand physiological data of children with ADHD.

Additional research should be undertaken to examine how smartwatch based DHI might support children with ADHD and their families more broadly. Currently, our DHI only works on iOS devices. However, other operating systems and devices should be explored to make a more accessible and affordable solution (e.g., Android, Fitbit). This pilot study shows that a set of functionalities, which are also available on other wearable devices (e.g., timed reminders), are useful for children with ADHD.

In particular, improving self-regulatory skills involves targeting specific contexts and personalizing interventions [81]. This kind of targeting is only possible by modeling the context during which people receive health information, including their physiological and mental states and the surrounding environment. These models do not yet exist, and their development would be a substantial contribution to both behavioral science and computing research. Clinicians generally rely on clients to self-report on these dimensions, which can be unreliable. Similarly, for children participating in psychological interventions, providers often rely heavily on parent and teacher ratings of observed impairment, these observations occur in different contexts where different demands are being asked of the child. Thus, our future research will enable DHI to collect self-report data alongside sensor data for creating machine-based models to support personalized delivery of DHI.

## Conclusion

The results of this six-week pilot field study are encouraging and indicate that the continued development of applications for wearable devices aimed to support children and adolescents with ADHD at home as well as in educational settings and psychosocial interventions is warranted. Results around the themes of improved self-awareness and self-motivation are particularly relevant for this group who inherently struggle with skills of executive function and self-regulation, which often adversely impacts parent-child relations during childhood, adolescence, and young adulthood. While this pilot study was limited to a small group of participants in a convenience sample, it reveals a meaningful reflection of parents’ perceptions about supportive technology under exceptional circumstance—that quite likely, is representative of many more families experiencing the fall-out of this global crisis. This spontaneous pilot provided timely and rich information about how wearable device applications and DHI more broadly may help those children and adolescents most likely to experience difficulty with distance learning circumstances for the unknown duration of the COVID-19 pandemic.

## Supporting information

S1 File Interview protocol. (PDF)
