# Supplementary material for: Parents’ perspectives on a smartwatch intervention for children with ADHD: Rapid deployment and feasibility evaluation of a pilot intervention to support distance learning during COVID-19
Source: PLoS One. 2021 Oct 27;16(10):e0258959. doi: 10.1371/journal.pone.0258959 (PMC8550607; doi:10.1371/journal.pone.0258959)
Supplement: S1 File — (PDF) [file pone.0258959.s001.pdf]

Cool Craig Application
Parent Interview Protocol

Duration 30 min
6-weeks of use of watch/phone

Aim

- Understand the use and adoption of the smartwatch
- Understand the potential impact of the smartwatch on self-regulation
- Expectations for the goals/rewards dynamic (next stage of the study)

Introduction

Good afternoon, my name is ***[say your name]*** and this is ***[say interview partners name]***. We are both researchers in the Department of Informatics at the University of California, Irvine (UCI). We want to thank you for taking the time to speak with us today.

Today we will be conducting an interview. The **aim of this interview** is to understand how your child used the smartwatch. We want to understand the potential impact of smart watches on self regulation skills. We want to remind you that this is an evaluation for the smartwatch and not of you nor your child.

So, please feel free to tell us what you like about the watch, and what you did not like about the watch. Also, we are interested in hearing about what did not work as you had expected, and your overall opinion on such technology use for children. All of this information is very important as we can learn a lot from your feedback and we can use it to try to improve parent and child experience in the future. Feel free to ask us any questions or concerns at any time during this interview.

At the end of this interview, we will give you a brief introduction to the next stage. We would like to record this session, only the audio file will be kept and the video will be deleted immediately afterward. If you consent to having this session recorded please state, “I give consent to have this interview recorded.”

Opening

1. Could you explain to me when your child usually wears his watch?
2. How do you think your child feels about wearing the watch?

Expectations

3. How familiar were you with smartwatches prior to this study?
    a. What were the concerns you had regarding the watch?
    b. What were your concerns regarding the phone?
4. What were some expectations you had about the watch? (Example of expectations: improve organization skills, improve physical activity)

a. Do you think those expectations have met yet? Why? How?

Use and Adoption

5. How do you think your child felt about smartwatches before s/he got the watch?
6. What happened when s/he received it?
7. How do you think s/he feels now?
    a. (In case the children change their expectations)
        i. What do you think led to that change in attitude?
8. How would you describe your engagement with the watch or the iPhone?
    a. What would you say your role was in the use of the watch?
    b. How do you feel about serving in that role (i.e., is it what they expected, or does it feel like an extra task)?

9. What did you think is the most useful part of the smartwatch for you / for your kid?
    a. What makes this especially useful?
10. What did you think is the least useful part of the smartwatch for you / for your kid?
    a. How did this impact your idea of the watch?
    b. How did you deal with this less than useful quality/feature of the watch?

Potential Impact of the Smartwatch on ADHD Symptoms

11. In what ways do you believe a smartwatch affected the behavior of your child? Do you believe the watch has an effect on your child's behavior?
    a. What made this especially useful?
    b. How would you have liked the smartwatch to have worked to avoid this?

12. In what ways do you believe a smartwatch affects your child's ability to pay attention or manage his behavior?
    a. What made this especially useful?
    b. How would you have liked the smartwatch to have worked to avoid this?

13. How familiar are you with the concept of self-regulation? ***(have a table or graph ready for them)***
    a. In what ways do you believe a smartwatch has the potential to assist a child in developing and maintaining self-regulatory skills?
    b. Do you believe the watch assisted your child in maintaining any of the following regulation skills?

14. In what ways do you believe a smartwatch affected your child's organization skills?
    a. What made this especially useful?
    b. How would you have liked the smartwatch to have worked to avoid this?

15. In what ways do you believe a smartwatch affects your child's physical activity?
    a. What made this especially useful?
    b. How would you have liked the smartwatch to have worked to avoid this?

Functionality

16. Calendar
    a. How often do you find that you have to remind your child of their schedule?
    b. How do you think the watch affects your child's level of independence?
        i. Is your child more likely to adhere to a task if the watch reminds them to do it?
17. Physiological measurement
    a. Did you notice any comments or behaviors of your child in response to the measurements given by the watch (e.g., heart rate, sleep, physical activity)?
    b. Did you check on the iPhone the results of the physiological measurements on the watch?
        i. If yes, which ones? Do you find the information useful/understandable?
        ii. If not, why?

Next Steps of the Project

18. What is your involvement in the management of the watch and your child?
19. If possible, would you be interested in interaction with your child and the watch?
20. How would you envision yourself having more interaction with your child through the watch?
    a. What features do you wish the watch had in assisting you in caring for your child and supporting their growth?

In the next step of the project, we are going to install an app to the phone where you can add daily or weekly goals to your child's watch, so s/he can see them on the watch and select those that s/he thinks s/he accomplished. Then on the phone, you can give them "tokens" or points if they successfully accomplished the goals. The tokens can then be exchanged for rewards that you add on the phone app. More details and tutorials will be given to you soon. The app will be designed to support and reinforce the behavioral program of The Children's School and will be consistent with the parent training and child social skills and behavior curriculum. Our goal is to make it easier for families to implement the behavior program at home.

21. Are you still interested in continuing the project?
22. Would you like us to add a specific application to the watch?
